# Detection, Transmission, and Characterization of Grapevine Virus H in Croatia

**DOI:** 10.3390/pathogens10121578

**Published:** 2021-12-03

**Authors:** Martin Jagunić, Boris Lazarević, Kristina Nikolić, Domagoj Stupić, Darko Preiner, Darko Vončina

**Affiliations:** 1 Department of Plant Pathology, Faculty of Agriculture, University of Zagreb, 10000 Zagreb, Croatia; mjagunic@agr.hr; 2 Department of Plant Nutrition, Faculty of Agriculture, University of Zagreb, 10000 Zagreb, Croatia; blazarevic@agr.hr; 3 Centre of Excellence for Biodiversity and Molecular Plant Breeding (CroP-BioDiv), 10000 Zagreb, Croatia; dpreiner@agr.hr; 4 Faculty of Agriculture, University of Zagreb, 10000 Zagreb, Croatia; k.nikolic989@gmail.com; 5 Department of Viticulture and Enology, Faculty of Agriculture, University of Zagreb, 10000 Zagreb, Croatia; dstupic@agr.hr

**Keywords:** RT-PCR, vine mealybug, coat protein, RdRP, sequencing

## Abstract

A survey of recently discovered vitiviruses was performed on 113 Croatian autochthonous grapevine cultivars from the national collection “Jazbina” using one-step RT-PCR. The presence of grapevine virus H (GVH) was confirmed in nine (7.9%) cultivars and grapevine virus G in eight (7.1%), while the presence of grapevine viruses I and J were not detected. GVH was transmitted by the vine mealybug (*Planococcus ficus*) from a source plant to grapevine seedlings with a 10.5% transmission rate using a combination of 10 first and second instars per plant with 48 and 72 h of acquisition and inoculation access period, respectively. Transmission correlated with the presence of grapevine leafroll-associated virus 3 (GLRaV-3) in the GVH-source plant and recipient seedlings. No alternative GVH host was identified. A comparison of 356 nt fragments of the RdRP and CP coding regions showed nucleotide identity between the Croatian GVH isolates in the range of 95.5–99.2% and 97.5–99.4% and amino acid identity between 95.8 and 100% and between 98.3 and 100%, respectively. Comparison with foreign isolates revealed nucleotide sequence similarity in the RdRP and CP between 94 and 100% and between 97.7–100%, respectively. To the best of our knowledge, this is the first report of GVH in Croatia and the first identification of the vine mealybug as a vector of GVH.

## 1. Introduction

Grapevine (*Vitis vinifera* L.) is one of the most widely distributed woody plants, the products of which are extensively used for direct or indirect consumption. Croatia, as a country with a dual climate (Continental and Mediterranean) suitable for grape production, is characterized by a large number of autochthonous cultivars. In the pre-phylloxera period, more than 400 grape cultivars were cultivated [1], and today, around 125 autochthonous cultivars are known in Croatia [2], with increasing interest from local producers. In the last decade, several projects have been launched in various regions of Croatia for the revitalization, protection, and dissemination of these cultivars, including efforts of clonal and sanitary selection [1,3].

Viral diseases are considered important threats to cultivated crops, especially for those that are vegetatively propagated. The long viticultural tradition and exchange of planting material probably played an important role in the large number of different viruses infecting grapevines globally. In the case of Croatian viticulture, multiple viruses have been reported in autochthonous cultivars [4,5,6], especially those from the leafroll and rugose wood complexes, which are considered economically important [7,8]. 

In the last decade, the improvement of detection methods, in particular high-throughput sequencing (HTS), has confirmed the presence of new virus species in grapevine. In the period from 2016 to 2020 alone, 28 different viruses were reported using HTS. Among them, six new members of the genus *Vitivirus* were reported: grapevine virus G (GVG) and grapevine virus I (GVI) in Chardonnay from New Zealand [9,10]; grapevine virus H (GVH) from an unknown grapevine variety in Portugal [11]; grapevine virus J (GVJ) in the plant of cultivar ‘Kisil Sapak’ originating from Turkmenistan, but in a vine which was part of the Foundation Plant Services collection (FPS, UC -Davis, CA) [12]; grapevine virus L (GVL) in vines from Croatia, Canada, New Zealand, and China [13]; and grapevine virus M (GVM) from the hybrid Blanc du Bois in Texas [14]. The first discovered members of the genus *Vitivirus*, grapevine virus A (GVA) from grapevines in Italy showing symptoms of a pitted stem [15] and grapevine virus B (GVB) from grapevines with corky bark from Europe and North America [16], are known to be members of the so-called rugose wood complex and, depending on the rootstock/scion combination and climatic conditions, negatively affect grapevine production [17,18,19]. In addition to transmission by contaminated planting material, vector-mediated transmission by various species of mealybugs (*Pseudococcidae*) and scale insect (*Coccidae*) has been documented for GVA [20,21,22,23,24,25,26,27], GVB [16,21,27,28], and GVE [29], with no data available for the other members of the genus infecting grapevines. Several studies [26,27] have reported synergy in vector transmission between vitiviruses and viruses from the leafroll complex (particularly grapevine leafroll-associated viruses 1 and 3, GLRaV-1, and GLRaV-3), with vitiviruses being rare in single infection and an increased incidence and vitivirus titers in grapevines coinfected with leafroll viruses [30,31]. 

In addition to species of the genus *Vitis* as natural hosts, experimental transmission of vitiviruses to various herbaceous hosts (mainly the *Nicotiana* and *Chenopodium* species) by various insect vectors or mechanical inoculation has been documented for GVA and GVB [16,21,26,32,33,34,35].

The aim of this study was to obtain additional information on the distribution of some of the recently reported vitiviruses, with particular attention to the distribution and genetic variability of GVH in Croatia and its relationship with isolates from other countries, based on the partial replicase (RdRP) and coat protein (CP) gene sequences. In addition to genome information that could be useful for evolutionary processes and reliable detection methods, the study provided evidence of GVH transmission by vine mealybug (*Planococcus ficus*) and virus host range, as well as a possible link between GVH transmission and other viruses. This information is important for the development of control strategies in vineyards and prevention of pathogen dissemination. 

## 2. Results

### 2.1. Virus Detection

Virus screening for the presence of different vitiviruses (GVG, GVH, GVI, and GVJ) done on 113 different genotypes/cultivars from the national collection of autochthonous Croatian grapevine cultivars by one-step reverse transcription PCR (RT-PCR) confirmed only the presence of GVG and GVH in eight and nine genotypes/cultivars, respectively (Table 1). Nine GVH-positive vines (cvs. Gustopupica, Malvazija istarska, Muškatel, Babica plosnata, Brajdica bijela, Plavčina, Bljuzgavac, Svrdlovina crna, and Kozjak) resulted in RT-PCR products of a 400-base-pair size for both RdRP and CP regions (Supplementary Figure S1). All GVH-infected cultivars were from the coastal region of Croatia, except cv. Kozjak, which was from the continental Croatian wine-growing region (Supplementary Figure S2).

For the purpose of vector transmission experiments and selection of the most appropriate virus source, all GVH-positive vines were additionally tested for the presence of ArMV, GFLV, GLRaV-1, GLRaV-3, and GVA using quantitative PCR (qPCR). qPCR results revealed that all GVH-positive cultivars were coinfected with GLRaV-3 and GVA, three cultivars were coinfected with GLRaV-1, three with GVG, and one with ArMV (Table 2). 

### 2.2. Virus Transmission

According to the GVH screening conducted in the national collection “Jazbina”, the grape cv. Malvazija istarska was selected as a GVH source plant for transmission trials, as it was a cultivar with one of the lowest number of other viruses in coinfection (only GLRaV-3 and GVA, Table 2). After the 48 h AAP on 30 cm long shoots of Malvazija istarska, 10 first and second instars of vine mealybug (*Pl. ficus*) were transferred to the virus-free recipient plants for 72 h IAP. Three months later, of all recipient plants used in this survey, only two out of nineteen (10.5%) grapevine seedlings of cv. Žlahtina were positive for GVH with both primer sets (RdRP and CP). Further analysis of the two GVH-infected seedlings confirmed coinfection with GLRaV-3, but not with GVA. In both seedlings, Sanger sequencing revealed an identical GVH nucleotide sequence in the RdRP and CP gene sections as in the source plant Malvazija istarska (data not shown). No alternative GVH host was identified by RT-PCR among the other plant species used in this survey. However, high mortality of instars was observed in *N. benthamiana*, so this result should be taken with caution and validated in following seasons. 

### 2.3. Sequencing and Phylogenetic Analyses

**Figure 1:**
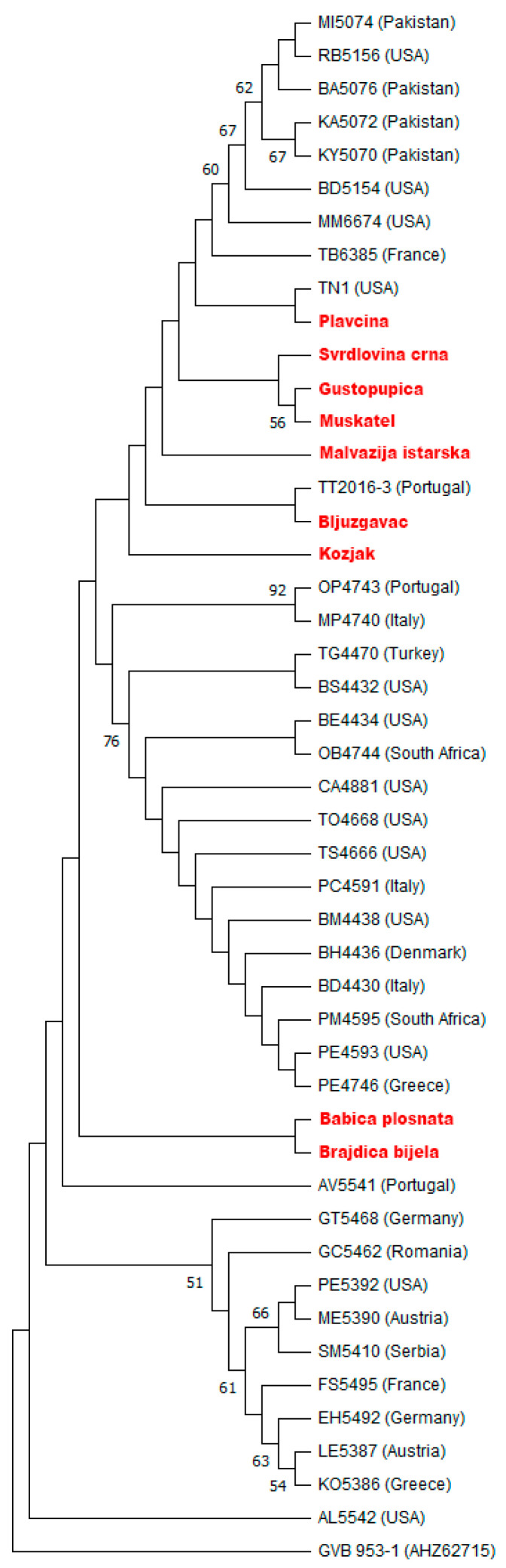
Maximum Likelihood (ML) tree showing phylogenetic relationships based on a 356 nts long sequences of the replicase (RdRP) coding region of 9 Croatian and 37 foreign isolates of grapevine virus H (GVH) reported worldwide. The ML tree was constructed using MEGA 11 with the Kimura 2-parameter + Gamma distribution (K2 + G) model of nucleotide substitution and GVB isolate 953-1 as the outgroup. Bootstrap values of 1000 replicates greater than 50% are shown at the tree nodes. Croatian GVH isolates are highlighted in red and represented by the corresponding cultivar names, while foreign isolates are denoted by the isolate names and their country of origin.

Sanger sequencing results for the 356 nts portion (corresponding to PCR products of 400 nts, excluding primers) of the RdRP gene for nine Croatian GVH isolates consisted of 331 conserved, 25 variable, and 9 parsimony-informative sites, with their nucleotide and amino acid identities ranging from 95.5 to 99.2% and from 95.8 to 100%, respectively. The aforementioned nucleotide variability resulted in nine amino acid differences (Supplementary Figure S3). In comparison with 37 GVH isolates reported from other parts of the world and the corresponding origin of their sources (Supplementary Table S1), estimates of evolutionary divergence between sequences showed identity ranging from 94 to 100% at the nucleotide level and from 93.9 to 100% at the amino acid level (Supplementary Figure S4). Phylogenetic analyses done through a maximum likelihood (ML) tree positioned Croatian GVH isolates, along with foreign isolates, as showing very low branch support for their eventual separation into different groups (Figure 1). 

**Figure 2:**
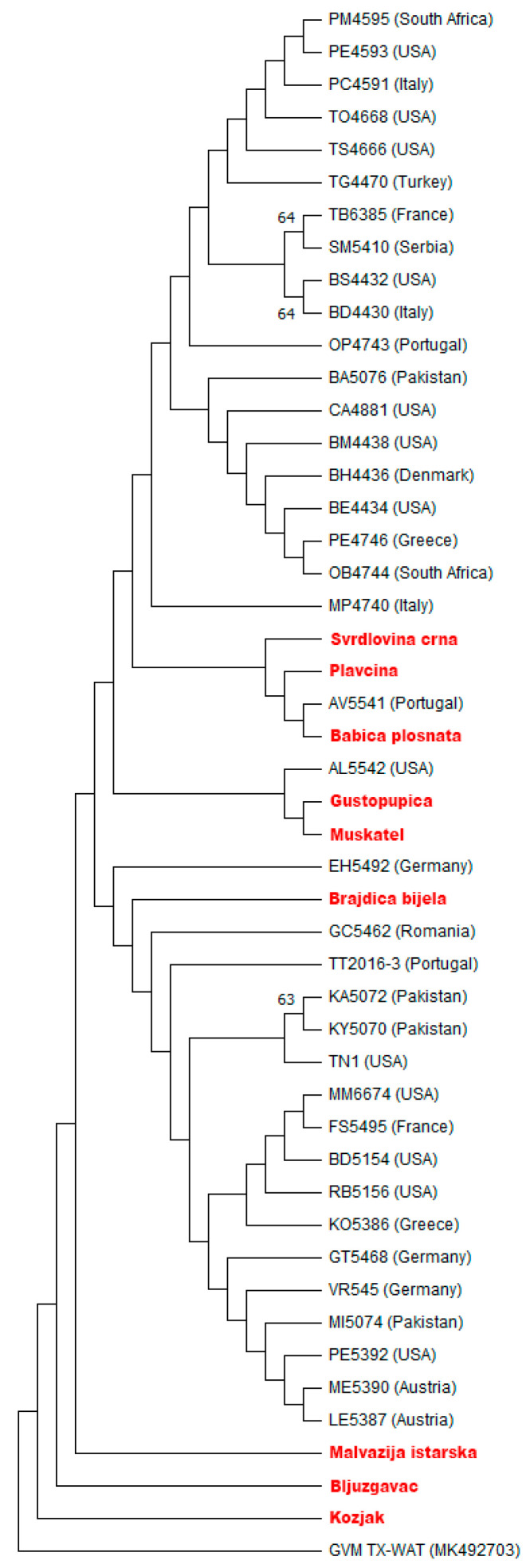
Maximum Likelihood (ML) tree showing phylogenetic relationships based on a 356 nts long sequences of the coat protein (CP) coding region of 9 Croatian and 38 foreign isolates of grapevine virus H (GVH) reported worldwide. ML tree was constructed using MEGA 11 with the Kimura 2-parameter + Gamma distribution (K2 + G) model of nucleotide substitution and GVM isolate TX-WAT as the outgroup. Bootstrap values of 1000 replicates greater than 50% are shown at the tree nodes. Croatian GVH isolates are highlighted in red and represented by the corresponding cultivar names, while foreign isolates are denoted by the isolate names and their country of origin.

Compared to the RdRP region, the CP region of Croatian GVH isolates was less divergent with 339 conserved, 17 variable, and 5 parsimony informative sites, with their nucleotide and amino acid identity ranging from 97.5 to 99.4% and from 98.3 to 100%, respectively. The mentioned nucleotide variability resulted in two differences at the amino acid level (Supplementary Figure S5). In comparison with 38 GVH isolates from other countries (Supplementary Table S1), sequences showed high nucleotide identity ranging from 97.7 to 100% at the nucleotide level and from 97.4 to 100% at the amino acid level (Supplementary Figure S6). Phylogenetic analyses performed using the maximum likelihood (ML) tree showed a similar situation as in the RdRP phylogenetic tree, with very low support for the formation of separate groups, so that the Croatian GVH isolates, with the exception of isolates Malvazija istarska, Bljuzgavac, and Kozjak, were scattered within the GVH isolates reported from other countries (Figure 2). 

All nine GVH sequences identified in this study in the RdRP and CP regions were submitted to GenBank as accession numbers OK474813-21 for RdRP and OK474822-30 for the CP region.

## 3. Discussion

The present study documents the occurrence of GVH in Croatian autochthonous grapevine cultivars as the eighth vitivirus confirmed in Croatia. After the discovery of GVH in a symptomless grapevine of an unknown cultivar in Portugal [11], in several cultivars at the USDA National Clonal Germplasm Repository (NCGR) in Winters [36], and recently in commercial vineyards in the cultivar Assyrtiko in Greece [37], this study extends the information on the GVH geographic distribution. Like the NCGR results, where GVH was confirmed in different cultivars from different viticultural regions, a similar situation was found in this study, as GVH was confirmed in cultivars mainly originating from three different sites located in the coastal part of Croatia with a Mediterranean climate (Supplementary Figure S2). Only cv. Kozjak was detected as a GVH source from the continental region outside of a Mediterranean climate.

In addition to GVH, the presence of another recently discovered vitivirus, GVG, was confirmed in eight vines, including three vines coinfected with GVH. The presence of GVG has already been reported from Croatia using HTS from several autochthonous cultivars, though originating from another collection [38]. These findings, together with the results of this study, suggest that GVG could be a widespread virus, especially in the coastal region.

The possibility of vector transmission, first suspected in the national collection “Jazbina” in the grapevines cv. Gustopupica and Muskatel, as two GVH-infected vines positioned next to each other, was confirmed during greenhouse transmission trials using first and second instars of the vine mealybug (*Pl. ficus*), a common pest in commercial vineyards worldwide, including Croatia. First and second instars of *Pl. ficus* proved to be capable of grape-to-grape transmission of GVH at a rate of 10.5% using 10 larvae per plant and 48 h of AAP and 72 h of IAP. Although experimental transmissions, either by mechanical inoculation or by insect vectors, have been documented for GVA and GVB [16,21,26,32,33,34,35], this study did not confirm that the herbaceous species studied can be infected with GVH using vine mealybug. So far, within vitiviruses, *Pl. ficus* has been described as a grape-to-grape vector of GVA [23,39,40] and GVB [16,28]. Because no GVH-only source plant was found during the screening process in the collection “Jazbina”, cv. Malvazija istarska coinfected with GLRaV-3 and GVA was used as a GVH source for the transmission experiments. Since the presence of GLRaV-3 but not GVA was confirmed by qPCR in both GVH-infected grapevine seedlings of cv. Žlahtina, there might be a link between the simultaneous transmission of GVH and GLRaV-3 where the latter could serve as a helper virus. This type of assisted transmission between viruses from the leafroll and rugose wood complexes has been suggested and documented by several studies [20,22,30,31]. Since GVA was not confirmed in infected grapevine seedlings at the time of testing, this suggests that transmission by *Pl. ficus* could be a useful tool for separation of simultaneous infections with different vitiviruses.

The genetic variability of the Croatian GVH isolates in the RdRP and CP regions determined in this study might be related to the different origin of the mother plants whose buds were used for grafting in the national collection “Jazbina”. An exception could be the isolates Gustopupica and Muskatel, adjacently positioned in the collection, suggesting a possible vector transmission. A possibility for vector transmission in this case can be justified with the sequencing results, which confirmed those two isolates as most similar with the highest nucleotide identity in the RdRP and CP regions (99.2% and 99.4%, respectively; Figure 1 and Figure 2).

Compared to GVH isolates reported worldwide, phylogenetic analyses were eventually not informative enough for separating into different groups/subgroups and formed groups with relatively low branch support. The partial CP region of Croatian isolates proved to be less divergent compared to the RdRP region. Since detection of GVH is now based exclusively on molecular methods, we believe that the data obtained in this study will contribute to the development of more accurate detection methods covering a larger number of different GVH strains.

The results of this study confirm the presence of GVH in Croatia and extend the knowledge about its geographic distribution. In addition to new data on its genome, this study partially fills the information gap regarding its transmission by vine mealybug and possible interactions with other viruses. In the era of high-throughput sequencing, which has led to the discovery of the genome of a considerable number of new grapevine viruses, such data are not only of scientific, but also of practical value.

## 4. Materials and Methods

### 4.1. Plant Material and RNA Extraction

The study was conducted on the national collection of Croatian autochthonous grapevine cultivars located in the experimental station “Jazbina”, managed by the Department of Viticulture and Enology (University of Zagreb Faculty of Agriculture). The collection includes 113 different genotypes/cultivars, each represented by 4 to 6 vines. Since the vines of the same cultivar were established using buds from the same mother plant and certified virus-free rootstocks, only one vine per cultivar was selected for virus-screening, assuming all vines from the same cultivars had the same virus status. Leaf samples were collected from one selected vine of each cultivar in May 2020 and RNA was isolated from 0.1 g of leaf petioles homogenized in a mortar with pestle and the addition of liquid nitrogen. Each sample was diluted with 1.8 mL of grinding buffer (0.015 M Na_2_CO_3_, 0.035 M NaHCO_3_, 0.0005 M PVP 40, 1 g/500 mL bovine serum albumin, 0.25 g/500 mL Tween 20; pH 9.6) and placed in a 2 mL collection tube. After centrifugation at 13,200 rpm for 10 min, 4 µ of the supernatant was mixed with 50 µL of GES (0.1 M glycine, 0.05 M NaCl, 0.001 M EDTA, 0.5% Triton X, 1% β-mercaptoethanol; pH 9.0) and denatured at 95 °C for 10 min in a Mastercycler (Eppendorf, Hamburg, Germany). The quality and quantity of isolated RNA were determined spectrophotometrically using a NanoPhotometer P330 Spectrophotometer (Implen, München, Germany).

### 4.2. Virus Detection

Vines from the national collection were tested for the presence of several vitiviruses: GVG, GVH, GVI, and GVJ. For this purpose, the one-step RT-PCR kit (Qiagen, Hilden, Germany) was used in a 25 µL reaction volume containing 0.5 µM of each primer, 2 µL of isolated RNA, and all other components according to the manufacturer’s instructions. For the detection of GVG, the primer pair F12CP/R12CP was used [38], while the confirmation of GVH, GVI, and GVJ was performed with primer pairs targeting the RdRP and CP regions [36]. RT-PCR was performed in one step using a Mastercycler (Eppendorf, Hamburg, Germany) under the following conditions: reverse transcription 30 min at 59 °C, initial activation step 15 min at 95 °C; 35 cycles of 30 s at 94 °C, 45 s at 55 °C, 1 min at 72 °C; and a final elongation step of 7 min at 72 °C. Visualization of PCR products was performed on a 2% agarose gel prepared in a 1XTBE buffer containing one drop of GelRed (CareDx AB, Stockholm, Sweden).

For the transmission experiments, GVH-positive grapevines were additionally tested by quantitative RT-PCR (RT-qPCR) for grapevine leafroll-associated viruses 1 and 3 (GLRaV-1, GLRaV-3) [41,42], grapevine virus A (GVA) [43]; arabis mosaic virus (ArMV), and grapevine fanleaf virus (GFLV), with primers and probes designed at Foundation Plant Services, UC Davis (personal communication). RT-qPCR was performed on a 7500 Real Time PCR System (Applied Biosystems, Thermo Fischer Scientific, Waltham, MA, USA). 

### 4.3. Virus Transmission

Transmission experiments were conducted under greenhouse conditions with vine mealybug larvae (*Pl. ficus*), a pest commonly found in Croatian vineyards. To ensure virus-free status of the vector colony reared on butternut squash, *Cucurbita moschata* Duchesne was used. The determination of the mealybug species was done by PCR [44]. As a GVH source, 30 cm long shoots of cv. Malvazija istarska were taken from the national collection and placed in water to maintain turgor pressure. As recipient plants, the following species were used: grapevine seedlings of cv. Žlahtina (19 plants), *Chenopodium murale* (14), *Abuthilon theophrasti* (3), *Amaranthus retroflexus* (4), *Ambrosia artemisifolia* (11), *N. benthamiana* (1), and *Papaver rhoeas* (1). Prior to transmission, all plants in the experiment were tested for the presence of GVH, GLRaV-3, and GVA using one-step RT-PCR or RT-qPCR, as previously described. Based on the results of vector transmission studies for other vitiviruses [27,40], a 48 h acquisition access period (AAP) and an extended time of 72 h inoculation access period (IAP) were used. After AAP on cv. Malvazija istarska, 10 instars were transferred to each of the recipient plant species. After the IAP, the instars were mechanically removed and the plants were sprayed with an insecticide (imidacloprid) and mineral oil (white oil). Three months later, the plants were tested for viruses present in the source plant of cv. Malvazija istarska using the previously described protocols.

### 4.4. Sequencing and Phylogenetic Analyses

RT-PCR products from GVH-positive cultivars from the national collection targeting part of the RdRP and the CP regions with an expected product size of 400 bps were Sanger-sequenced in both directions at Macrogen (Amsterdam, The Netherlands). Sequences were analyzed using the BioEdit 7.2 [45] and Mega 11 [46] programs. After primer removal, the products containing 356 nucleotides (consensus sequences) were used for cross-comparison and comparison with other GVH isolates whose sequences were deposited in the GenBank (Supplementary Table S1). Multiple sequence alignment was performed using Clustal X within the Mega 11 program. Best model of nucleotide substitution and the construction of phylogenetic trees using the maximum-likelihood method with 1000 bootstrap replicates was done using the Mega 11 program.

## Figures and Tables

**Table 1:** The presence of recently discovered vitiviruses in the national collection of Croatian autochthonous grapevine cultivars located in the experimental station “Jazbina”, determined by conventional one-step RT-PCR. Grapevine virus H (GVH)-infected vines are highlighted in yellow, grapevine virus G (GVG)-infected vines are highlighted in green, and GVH+GVG mixed infections are highlighted in red. The presence of other vitiviruses included in the study (GHI, GVJ) was not confirmed.

Cultivar	Cultivar	Cultivar	Cultivar
Pošip crni Maraština Dugovrst Babić Vranac Cibib Zlatarica vrgorska Crljenak kaštelanski 1 Crljenak kaštelanski 2 Ruža bijela II Palaruša hvarska Dobričić Grk Drnekuša mala Zadarka Ninčuša Kadarun Lasina Dišeča ranina Plavac mali sivi II Zinfandel Vlaški crljenak Medna Žilavka Žumić Muškat bijeli omiški Pavicić Palaruša viška Jarbola	Sušac Malvazija župska Bilan bijeli Beret Šemperinka Gustopupica Lelekuš Galac Stara brajda Bak Rudež Plavac mali crni Zlatarica blatska Gustopupica 0031 Kujundžuša Prč Ninska crvena Crljenak viški Malvazija dubrovačka bijela Šarica trišnjevica Trojščina Divjaka Malvazija istarska Moslavac Siložder Muškatel Bogdanuša Cipar Garganja	Lun Krstičevica Krivaja crvena Mijajuša Mekuja Cetinka Babica plosnata Plavac mali sivi Ruža bijela I Štajerka Marinkovića grozje Pribidrag Kadarka Debit Palagružonka Žlahtina Stradunska Silbijanac Topol Pršljivka Muškat ruža Frmentun Vranac Teran Bijeli debejan Plavec žuti Pošip Primitivo Glavanjuša	Brajdica bijela Petovka Gegić Drnekuša mala Oskorušica Kuć bijeli Razaklija Crnka Lipovina Plavčina Rogoznička Bratkovina crvena Šljiva Tanetova Bljuzgavac Debejan crni Kurtelaška Bratkovina bijela Svrdlovina crna Kozjak Mladenka Šipelj Svjetljak Glavinuša Babica Ljutun

**Table 2:** Grapevine virus H infected cultivars coinfected with other viruses (ArMV—arabis mosaic virus; GFLV—grapevine fanleaf virus; GLRaV-1, 3—grapevine leafroll-associated virus 1, 3; GVA—grapevine virus A; GVG—grapevine virus G).

Cultivar	Viruses				
	ArMV GFLV	GLRaV-1	GLRaV-3	GVA	GVG
Babica plosnata		+	+	+	
Bljuzgavac		+	+	+	+
Brajdica bijela	+		+	+	
Gustopupica			+	+	
Kozjak			+	+	+
Malvazija istarska			+	+	
Muškatel			+	+	+
Plavčina			+	+	
Svrdlovina crna		+	+	+	

## Data Availability

All sequencing data of Croatian GVH isolates obtained during the research were deposited in GenBank under accession numbers OK474813-21 for RdRP and OK474822-30 for CP region.

## Supplementary Materials

The following are available online at https://www.mdpi.com/article/10.3390/pathogens10121578/s1, Table S1: Grapevine virus H isolates used for comparison and phylogenetic analyses in replicase (RdRP) and coat protein (CP) regions with their origin and corresponding GenBank accession numbers. Figure S1: Agarose gel electrophoresis of RT-PCR products amplified with the one-step RT-PCR protocol using primers targeting the replicase (RdRP) and coat protein (CP) genes. Figure S2: The geographic origin of the GVH-positive grapevine cultivars introduced into the national collection “Jazbina”. Figure S3: Multiple alignment of the 356 nts and 118 aa region of the replicase (RdRP) coding sequences from nine Croatian grapevine virus H (GVH) isolates. Figure S4: Estimates of evolutionary divergence between Croatian GVH isolates and those from GenBank based on the 356 nts fragment of RdRP coding sequences using the p-distance (pairwise identity) model. Figure S5: Multiple alignment of the 356 nts and 118 aa region of the coat protein (CP) coding sequences from nine Croatian grapevine virus H (GVH) isolates. Figure S6: Estimates of evolutionary divergence between Croatian GVH isolates and those from the GenBank based on the 356 nts fragment of the CP coding sequences using the p-distance (pairwise identity) model.
