# Supplementary material for: Detection, Transmission, and Characterization of Grapevine Virus H in Croatia
Source: Pathogens. 2021 Dec 3;10(12):1578. doi: 10.3390/pathogens10121578 (PMC8704696; doi:10.3390/pathogens10121578)
Supplement: Supplementary file 1 [file pathogens-10-01578-s001.zip › Supplementary Figure S1_R1.pdf]

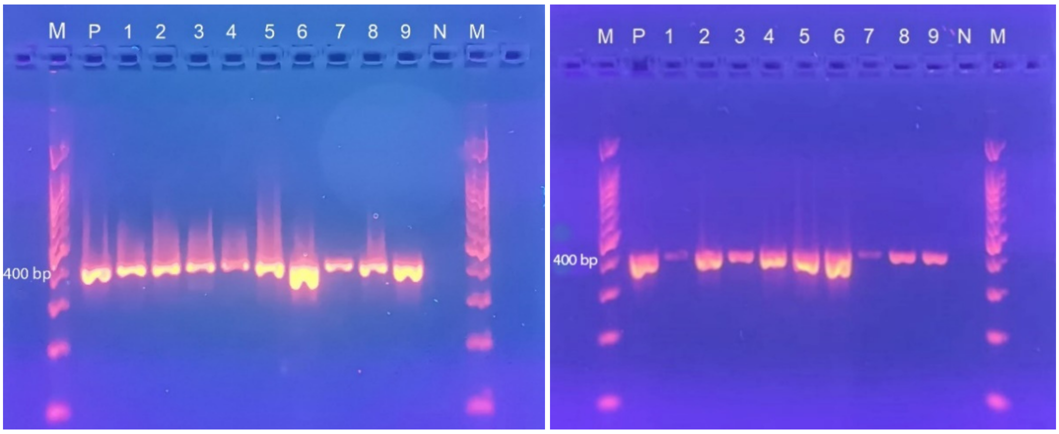

Supplementary Figure S1. Agarose gel electrophoresis of RT-PCR products amplified with the one-step RT-PCR protocol using primers targeting the replicase (RdRP - left) and coat protein (CP – right) genes. Expected product size in both cases: 400 base pairs (bp). M- DNA marker (GelPilot 100 bp Plus Ladder, Qiagen, USA), 1–9 samples (cvs. Gustopupica, Malvazija istarska, Muškatel, Babica plosnata, Brajdica bijela, Plavčina, Bljuzgavac, Svrdlovina crna and Kozjak, respectively), N - negative control/virus-free vine.
