# Supplementary material for: Detection, Transmission, and Characterization of Grapevine Virus H in Croatia
Source: Pathogens. 2021 Dec 3;10(12):1578. doi: 10.3390/pathogens10121578 (PMC8704696; doi:10.3390/pathogens10121578)
Supplement: Supplementary file 1 [file pathogens-10-01578-s001.zip › Supplementary Figure S2_R3.pdf]

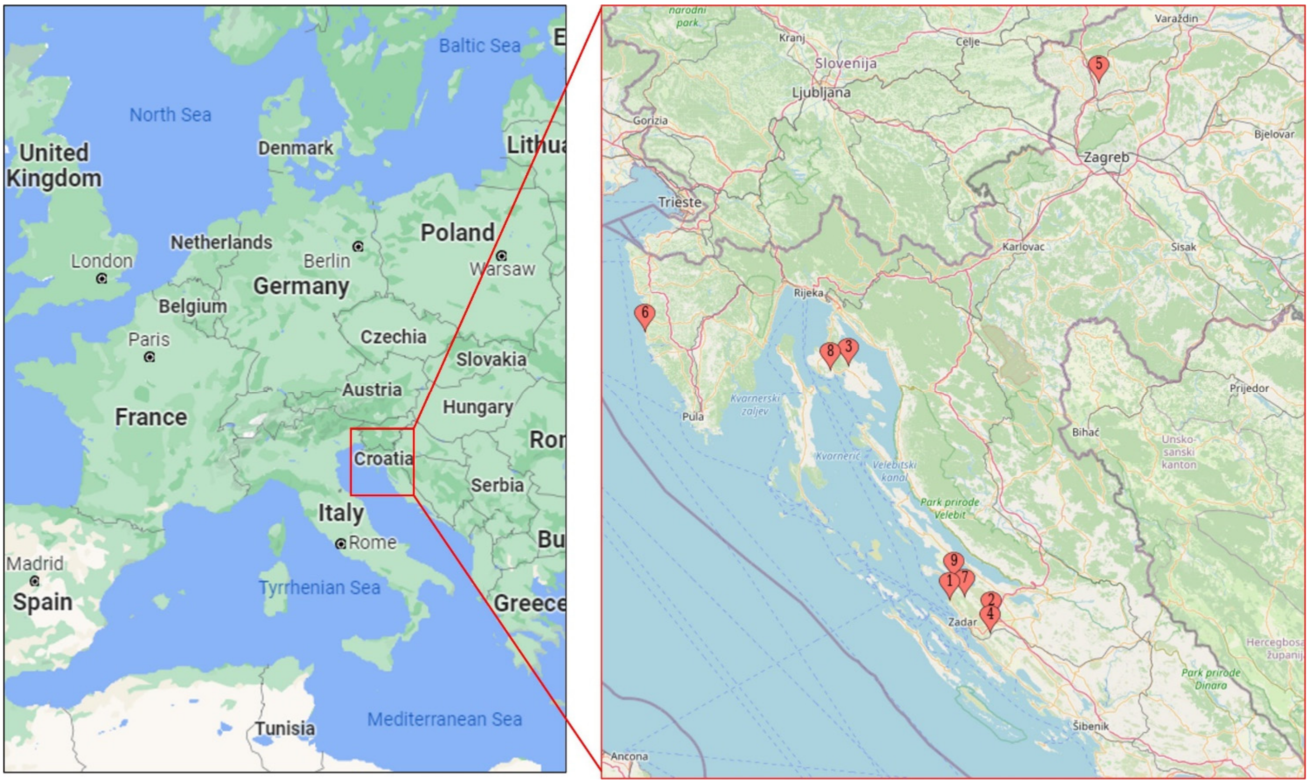

Supplementary Figure S2. The geographic origin of the GVH positive grapevine cultivars introduced into the national collection "Jazbina". The majority of the GVH positive cultivars originated from the Croatian coastal region and only one from the continental region (cv. Kozjak). Numbers on the map correspond to the following cultivars: 1 -Babica plosnata, 2 – Bljuzgavac, 3 - Brajdica bijela, 4 – Gustopupica, 5 – Kozjak, 6 - Malvazija istarska, 7 – Muškatel, 8 – Plavčina and 9 - Svrdlovina crna.
