# Supplementary material for: Detection, Transmission, and Characterization of Grapevine Virus H in Croatia
Source: Pathogens. 2021 Dec 3;10(12):1578. doi: 10.3390/pathogens10121578 (PMC8704696; doi:10.3390/pathogens10121578)
Supplement: Supplementary file 1 [file pathogens-10-01578-s001.zip › Supplementary Figure S4_R1.pdf]

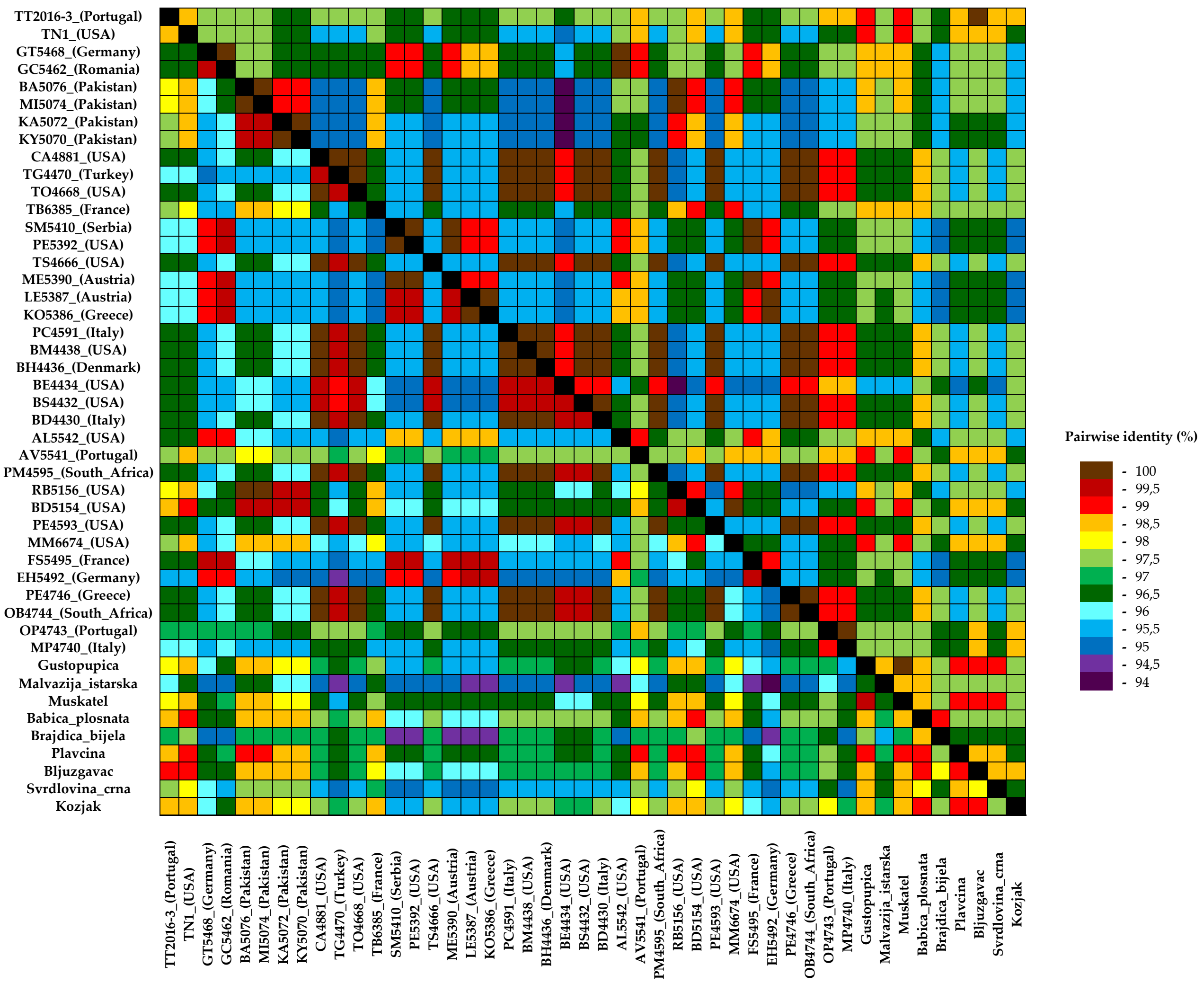

Supplementary Figure S4. Estimates of evolutionary divergence between Croatian GVH isolates and those from the GenBank based on the 356 nts fragment of the RdRP coding sequences using the p-distance (pairwaise identity) model: nucleotide level (below diagonal); amino-acid level (above diagonal). Croatian GVH isolates are represented by the corresponding grapevine cultivar names from which they were isolated.
