# Supplementary material for: Detection, Transmission, and Characterization of Grapevine Virus H in Croatia
Source: Pathogens. 2021 Dec 3;10(12):1578. doi: 10.3390/pathogens10121578 (PMC8704696; doi:10.3390/pathogens10121578)
Supplement: Supplementary file 1 [file pathogens-10-01578-s001.zip › Supplementary Figure S5_R1.pdf]

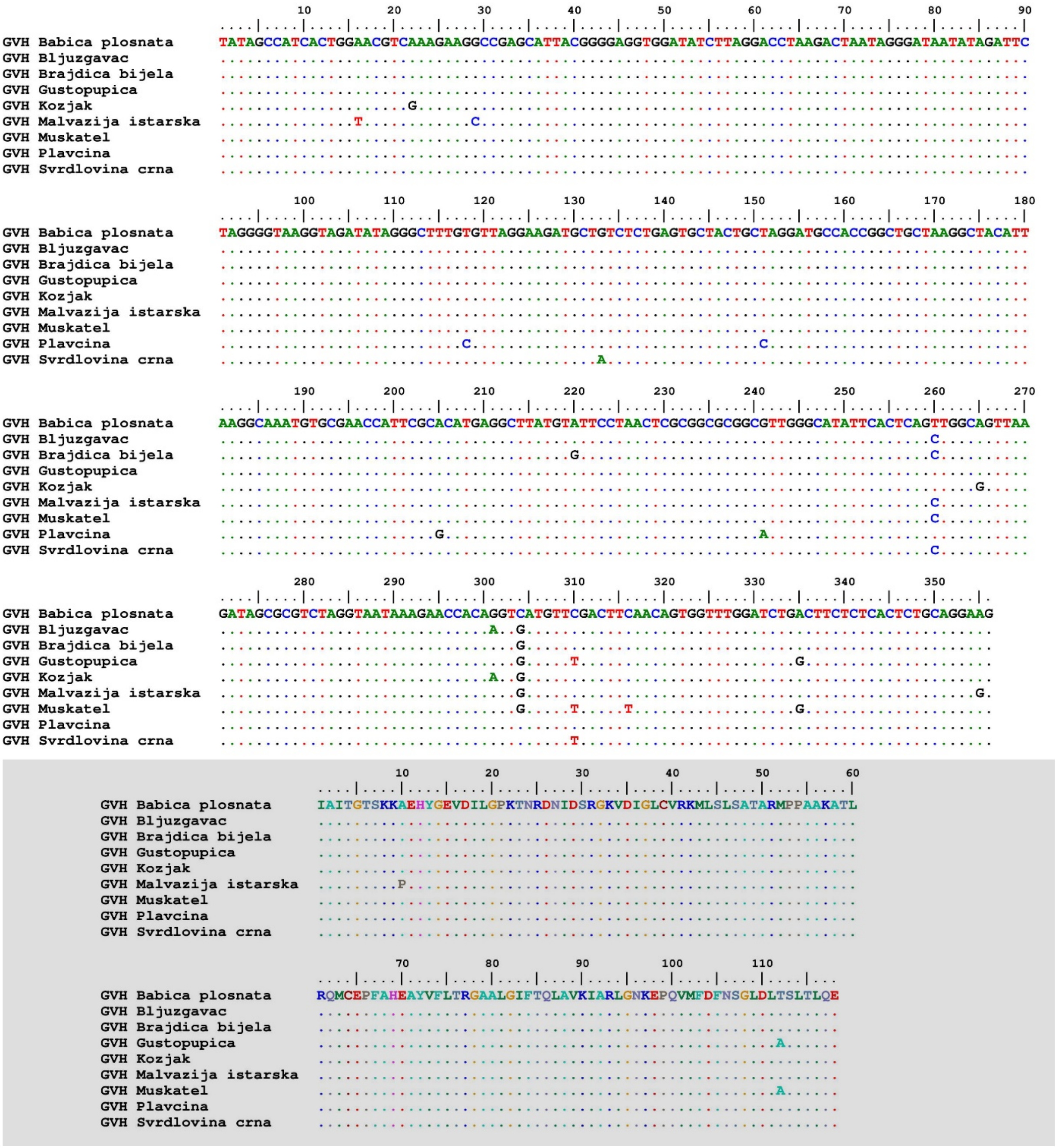

Supplementary Figure S5. Multiple alignment of the 356 nts (white) and 118 aa (grey) region of the coat protein (CP) coding sequences from nine Croatian grapevine virus H (GVH) isolates. The Croatian GVH isolates are represented by the corresponding grapevine cultivar names from which they were isolated.
