# Supplementary material for: Detection, Transmission, and Characterization of Grapevine Virus H in Croatia
Source: Pathogens. 2021 Dec 3;10(12):1578. doi: 10.3390/pathogens10121578 (PMC8704696; doi:10.3390/pathogens10121578)
Supplement: Supplementary file 1 [file pathogens-10-01578-s001.zip › Supplementary Table S1_R3.pdf]

Supplementary Table S1. Grapevine virus H isolates used for comparison and phylogenetic analyses in replicase (RdRP) and coat protein (CP) regions with their origin and corresponding GenBank accession numbers.

Isolate	Country	Cultivar	GenBank Acc. No.	
			RdRp	CP
TT2016-3	Portugal	/	NC_040545.1	NC_040545.1
TN1	USA	/	MN716768.1	MN716768.1
GT5468	Germany	Gamay Toma	MK017760.1	MK838928.1
VR5454	Germany	Vranac	/	MK838927.1
GC5462	Romania	Grassa de Cothari	MK017758.1	MK838926.1
BA5076	Pakistan	Bargoon	MK017757.1	MK838925.1
MI5074	Pakistan	Millishun	MK017756.1	MK838924.1
KA5072	Pakistan	Kabuli	MK017755.1	MK838923.1
KY5070	Pakistan	Kini Yatch	MK017754.1	MK838922.1
CA4881	USA	Canner	MK017753.1	MK838921.1
TG4470	Turkey	Turkish Grape	MK017752.1	MK838920.1
TO4668	USA	Tokay	MK017751.1	MK838919.1
TB6385	France	Terret blanc	MK017750.1	MK838918.1
SM5410	Serbia	Smederevka	MK017749.1	MK838917.1
PE5392	USA	Perruno	MK017748.1	MK838916.1
TS4666	USA	Tschilaci	MK017747.1	MK838915.1
ME5390	Austria	Mezes	MK017746.1	MK838914.1
LE5387	Austria	Leanyka	MK017745.1	MK838913.1
KO5386	Greece	Kokinostaphylo	MK017744.1	MK838912.1
PC4591	Italy	Perla di Csaba	MK017743.1	MK838911.1
BM4438	USA	Black Monukka	MK017742.1	MK838910.1
BH4436	Denmark	Black Hamburg	MK017741.1	MK838909.1
BE4434	USA	Bellino	MK017740.1	MK838908.1
BS4432	USA	Beauty Seedless	MK017739.1	MK838907.1
BD4430	Italy	Barone Dell'aterno	MK017738.1	MK838906.1
AL5542	USA	Aligote	MK017737.1	MK838905.1
AV5541	Portugal	Alicante Vermelho	MK017736.1	MK838904.1
PM4595	South Africa	Precoce de Malingre	MK017735.1	MK838903.1
RB5156	USA	Rish Baba	MK017734.1	MK838902.1
BD5154	USA	Black Damascus	MK017733.1	MK838901.1
PE4593	USA	Perlette	MK017732.1	MK838900.1
MM6674	USA	Morio Muscat	MK017731.1	MK838899.1
FS5495	France	Fer Servadou	MK017730.1	MK838898.1
EH5492	Germany	Ehrenfelser	MK017729.1	MK838897.1
PE4746	Greece	Petinos	MK017728.1	MK838896.1
OB4744	South Africa	Olivette Bauthelette	MK017727.1	MK838895.1
OP4743	Portugal	Olho de Pargo	MK017726.1	MK838894.1
MP4740	Italy	Maria Pirovano	MK017725.1	MK838893.1
